# Association between SARC-F scores and risk of adverse outcomes in older patients with cardiovascular disease: a prospective study at a tertiary hospital in the south of Vietnam

**DOI:** 10.3389/fmed.2024.1406007

**Published:** 2024-07-04

**Authors:** Tan Van Nguyen, Tuan Dinh Nguyen, Hung Cao Dinh, Tuan Dinh Nguyen, Trinh Thi Kim Ngo, Dung Viet Do, Thanh Dinh Le

**Affiliations:** ^1^Department of Geriatrics and Gerontology, University of Medicine and Pharmacy at Ho Chi Minh City, Ho Chi Minh City, Vietnam; ^2^Department of Interventional Cardiology, Thong Nhat Hospital, Ho Chi Minh City, Vietnam; ^3^Department of Internal Medicine, Faculty of Medicine, Pham Ngoc Thach University of Medicine, Ho Chi Minh City, Vietnam; ^4^Department of Internal Medicine, Faculty of Medicine, Nguyen Tat Thanh University, Ho Chi Minh City, Vietnam; ^5^Department of Medicine, Nguyen Tat Thanh University, Ho Chi Minh City, Vietnam

**Keywords:** sarcopenia, SARC-F questionnaire, cardiovascular disease, mortality, Vietnam

## Abstract

**Introduction:**

Older patients typically face elevated mortality rates and greater medical resource utilization during hospitalizations compared to their younger counterparts. Sarcopenia, serving as a prognostic indicator, is related to disability, diminished quality of life, and increased mortality. The SARC-F questionnaire, known for its cost-effectiveness, offers a valuable means of assessing sarcopenia. This study aims to explore the association between SARC-F scores and risk of adverse outcomes in elderly patients with cardiovascular disease at a Ho Chi Minh City hospital.

**Method:**

Participants aged 60 and above, admitted to the Department of Cardiology - Interventional and Cardiovascular Emergency of Thong Nhat Hospital in Ho Chi Minh City from November 2021 to June 2022, were recruited for the prospective, single-center study. The prognostic outcomes included all-cause death and the initial occurrence of emergency re-hospitalization within 6 months’ post-discharge. The Kaplan–Meier analysis compared the overall survival rates between different SARC-F score groups.

**Results:**

The study enrolled 285 patients with a median age of 74 (67, 81). During a 6-month follow-up period, there were 14 cases of mortality. A SARC-F score of 4 or higher was significantly associated with an increased risk of all-cause mortality, with HR of 2.02 (95% CI: 1.39–2.92, *p* < 0.001), and higher incidence of re-hospitalization events with RR of 1.66 (95% CI: 1.06 to 2.59, *p* = 0.026). Kaplan–Meier survival analysis indicated a notably higher mortality rate in the patients with high SARC-F scores (*p* < 0.001).

**Conclusion:**

In elderly patients with cardiovascular disease, the SARC-F questionnaire could serve as a simple and cost-effective method for detecting mortality and the risk of re-hospitalization.

## Introduction

As the global population continues to age, the healthcare system faces increasing challenges in managing the health and well-being of older individuals (1). Older patients often experience higher mortality rates and require more extensive medical resources during hospitalizations compared to their younger counterparts (2, 3). Therefore, it becomes essential to identify prognostic markers that can effectively forecast outcomes in this vulnerable demographic. Doing so is vital for enhancing patient care quality and optimizing the allocation of healthcare resources.

Sarcopenia, a progressive systemic skeletal muscle disease, is recognized as a key prognostic factor for elderly patients (4). This condition is strongly associated with adverse outcomes in elderly patients, including onset of disability (5), quality of life decline (6), and mortality (7). Besides, cardiovascular disease (CVD), a leading cause of morbidity and mortality in the elderly population, is particularly concerning in relation to sarcopenia (8).

The SARC-F questionnaire, consisting of five questions, offers a simple and cost-effective method for assessing sarcopenia (9). Additionally, the SARC-F has demonstrated a significant correlation between motor function and overall prognosis in individuals with CVD (10). This makes the SARC-F a valuable tool for the early detection of sarcopenia and for evaluating motor function and prognosis in patients with CVD.

While the SARC-F questionnaire has shown promise in identifying sarcopenia (11, 12), studies exploring its association with short-term prognosis remain limited. Understanding the relationship between SARC-F scores and the risk of adverse events can provide valuable insights into the prognostic value of this screening tool. Moreover, such knowledge can aid healthcare professionals in identifying high-risk patients and implementing appropriate interventions to improve outcomes. In Vietnam, the burden of CVD is increasing among older adults due to factors such as sedentary lifestyles, unhealthy diets, and an aging population demographic. Specific challenges faced by elderly patients with CVD in Vietnam include limited access to specialized cardiovascular care and a lack of awareness about cardiovascular risk factors and preventive measures among both patients and healthcare providers. Moreover, such knowledge can aid healthcare professionals in identifying high-risk patients and implementing appropriate interventions to improve outcomes. Therefore, this study aims to investigate the association between SARC-F scores and total combined events, comprising both all-cause death and all-cause re-hospitalization within 6 months’ post-discharge in elderly patients admitted to the Cardiovascular Department of a tertiary hospital in Ho Chi Minh City, Vietnam. By elucidating this association, the study seeks to provide valuable insights into optimizing care for elderly patients with CVD and improving their clinical outcomes in the Vietnamese healthcare context.

## Materials and methods

### Study design and participants

This prospective, single-center study comprised a cohort of 285 patients. The inclusion criteria for participant selection were individuals aged 60 years and above who were hospitalized at the Department of Cardiology - Interventional and Cardiovascular Emergency of Thong Nhat Hospital in Ho Chi Minh City from November 2021 to June 2022, including those discharged in this period. The exclusion criteria encompassed individuals with acute severe illness, those with a pacemaker implant, individuals incapable of completing the questionnaire and physical examination. Convenience sampling was employed for participant recruitment.

Ethical approval was obtained from the Ethical Committee of the University of Medicine and Pharmacy at Ho Chi Minh City (Number: 544/HĐĐĐ-ĐHYD, signed September 22, 2022). Informed consent was obtained from all participating individuals, and all research procedures were conducted in accordance with the relevant ethical guidelines and regulations.

### Parameters

In this study, we collected demographic information, body mass index (BMI), comorbidities, and laboratory test results. BMI was classified according to the World Health Organization guidelines, categorizing individuals as underweight (BMI < 18.5 kg/m^2^), normal weight (BMI 18.5–22.9 kg/m^2^), overweight (BMI 23–24.9 kg/m^2^), and obese (BMI ≥ 25.0 kg/m^2^) (13). The presence of comorbidities, including hypertension, coronary disease, heart failure, dyslipidemia, and diabetes, was identified either through diagnoses from specialists or extracted from patients’ medical records. Smoking was defined as either currently smoking or having quit within the past year. For assessing kidney function, the estimated glomerular filtration rate (eGFR) was calculated using the Cockcroft – Gault equation (14).

### Acquisition of SARC-F

Upon admission, patients underwent evaluation using the SARC-F questionnaire. This assessment involved interviewing for five key elements: strength, assistance in walking, ability to rise from a chair, capacity to climb stairs, and frequency of falls. Each component of the SARC-F was scored on a scale of 0 to 2 points, resulting in a cumulative score ranging from 0 to 10 points, with 0 representing optimal physical performance and 10 indicating the poorest. Strength was measured by asking, “How much difficulty do you have in lifting and carrying 10 pounds?” (None = 0; Some = 1; A lot or unable = 2). Assistance in walking was assessed by questioning, “How much difficulty do you have walking across a room?” (None = 0; Some =1; A lot, use aids, or unable = 2). The ability to rise from a chair was determined by, “How much difficulty do you have transferring from a chair or bed?” (None = 0; Some = 1; A lot or unable without help = 2). Climbing stairs was evaluated by, “How much difficulty do you have climbing a flight of 10 stairs?” (None = 0; Some = 1; A lot or unable = 2). Falls were assessed by, “How many times have you fallen in the past year?” (None = 0; 1–3 falls = 1; 4 falls = 2) (9). Patients with a total score of 4 or higher were classified as having sarcopenia (9).

### Prognostic outcomes

The prognostic outcomes in this study were established as a composite endpoint, encompassing all-cause mortality and the first occurrence of re-hospitalization. The assessment of patient prognoses was conducted over 6 months’ post-discharge. The duration until reaching the composite endpoint was determined by calculating the number of days from the patient’s discharge to the occurrence of the events, whether it be all-cause death or re-hospitalization.

### Statistical analysis

Categorical variables were described using frequencies and percentages (n (%)), while continuous variables were presented as mean with standard deviation (mean ± SD) or median with interquartile range (median (IQR)) according to the type of variables. To compare overall survival rates between two groups distinguished by the optimal SARC-F cut-off point for mortality prediction, Kaplan–Meier survival analysis alongside log-rank tests was employed. A multivariate analysis was conducted to identify factors influencing mortality, adjusting for age, existing morbidities, and nutritional status. Additionally, separate multivariate analyses were performed for each component of the SARC-F questionnaire to pinpoint specific items that significantly influence mortality. All statistical analyses were carried out using SPSS version 21 (IBM Japan, Tokyo, Japan), and a *p*-value of less than 0.05 was considered statistically significant.

## Results

### Demographic of study population

This research enrolled 285 participants aged 60 and above who completed the SARC-F questionnaire. Out of the total participants, 87 individuals (30.5%) were classified as having sarcopenia, indicated by a SARC-F score of 4 points or higher. The baseline characteristics of the study population is provided in Table 1. The cohort comprised 48.4% male and 51.6% female patients. The analysis revealed that those in the sarcopenia group were significantly older and women exhibited a higher prevalence of sarcopenia compared to men (*p* < 0.001). The participants’ average BMI was 22.8 ± 3.33 kg/m^2^, and nearly half of them (48.8%) were classified as overweight. Among the 285 patients, a high prevalence of hypertension was observed, with 88.1%, while 56.1% had coronary artery disease, and 22.1% suffered from heart failure. The cohort observed considerable rates of dyslipidemia and diabetes, with 57.9 and 40%, respectively. Additionally, a higher proportion of patients in the sarcopenia group had been diagnosed with heart failure (34.5% vs. 16.7%, *p* = 0.001) and had a greater prevalence of diabetes (50.6% vs. 35.4%, *p* = 0.016) than those in the normal group. Patients with sarcopenia had a higher rate of re-hospitalization within 6 months’ post-discharge compared to the robust ones (*p* = 0.001). Furthemore, the percentage of patients who experienced all-cause death was higher in patients with sarcopenia than in those without sarcopenia (*p* = 0.037).

**Table 1:** The baseline characteristics of patients.

Variable	Total (*n* = 285)	Sarcopenia (SARC-*F* ≥ 4) *N* = 87	Nonsarcopenia (SARC-*F* < 4) *N* = 198	*p*-value
Age (years)	74 (67, 81)	79.49 ± 7.88	71 (64, 78)	**<0.001**
Female, *n* (%)	147 (51.6)	63 (72.4)	84 (42.4)	**<0.001**
BMI (kg/m^2^)	22.8 ± 3.33	22.34 ± 3.21	22.95 ± 3.37	0.164
< 18.5	25 (8.8)	9 (10.3)	16 (8.1)	0.298
18.5–22.9	121 (42.5)	42 (48.3)	79 (39.9)	
23–24.9	71 (24.9)	21 (24.1)	50 (25.3)	
≥ 25.0	68 (23.8)	15 (17.3)	53 (26.7)	
Comorbidities				
Hypertension, *n* (%)	251 (88.1)	76 (87.4)	175 (88.4)	0.805
Coronary disease, *n* (%)	160 (56.1)	43 (49.4)	117 (59.1)	0.130
Heart failure, *n* (%)	63 (22.1)	30 (34.5)	33 (16.7)	**0.001**
Dyslipidemia, *n* (%)	165 (57.9)	45 (51.7)	120 (60.6)	0.162
Diabetes, *n* (%)	114 (40.0)	44 (50.6)	70 (35.4)	**0.016**
CKD, *n* (%)	36 (12.6)	12 (13.8)	24 (12.1)	0.696
Current smoking, *n* (%)	34 (11.9)	4 (4.6)	30 (15.2)	**0.010**
eGFR	72.3 (63.7–80.8)	72.3 (63.7–81.2)	72.3 (63.7–80.8)	0.661
All-cause re-hospitalization, *n* (%)	103 (36.1)	44 (50.6)	59 (29.8)	**0.001**
All-cause death, *n* (%)	14 (4.9)	8 (9.2)	6 (3.0)	**0.037**

BMI, Body mass index, CKD, Chronic kidney disease, *n*, Number. Significant p-values are presented in bold.

### SARC-F scores distribution

Table 2 illustrates the distribution of SARC-F scores, highlighting pronounced disparities between patients with and without sarcopenia. In the aspect of strength, participants in the sarcopenia group faced notable challenges, whereas individuals in the non-sarcopenia groups experienced minimal or no strength difficulties. This trend extended to mobility, where the sarcopenia group demonstrated a higher dependency on walking assistance, unlike their non-sarcopenia counterparts. Similarly, the sarcopenia group encountered more pronounced difficulties in rising from a chair and climbing stairs compared to the non-sarcopenia group. Moreover, the incidence of falls was significantly higher in the sarcopenia group than in those without this condition (*p* < 0,001).

**Table 2:** SARC-F score details.

Variable	Total (*n* = 285)	Sarcopenia (SARC-F ≥ 4) (*n* = 87)	Non-sarcopenia (SARC-F < 4) (*n* = 198)	*p-*value
Strength				
None (0)	185 (64.9)	12 (64.9)	173 (87.4)	**<0.001**
Some (1)	33 (11.6)	17 (11.6)	16 (8.1)	
A lot or unable (2)	67 (23.5)	58 (23.5)	9 (4.5)	
Assistance in walking				
None (0)	199 (69.8)	13 (14.9)	186 (93.9)	**<0.001**
Some (1)	76 (26.7)	64 (73.6)	12 (6.1)	
A lot or unable (2)	10 (3.5)	10 (11.5)	0 (0.0)	
Rise from a chair				
None (0)	211 (74.0)	26 (29.9)	185 (93.4)	**<0.001**
Some (1)	66 (23.2)	53 (60.9)	13 (6.6)	
A lot or unable (2)	8 (2.8)	8 (9.2)	0 (0.0)	
Climb stairs				
None (0)	106 (37.2)	2 (2.3)	104 (52.5)	**<0.001**
Some (1)	98 (34.4)	20 (23.0)	78 (39.4)	
A lot or unable (2)	81 (28.4)	65 (74.7)	16 (8.1)	
Falls				
None (0)	246 (86.3)	62 (71.3)	184 (92.9)	**<0.001**
Some (1)	34 (11.9)	20 (23.0)	14 (7.1)	
A lot or unable (2)	5 (1.8)	5 (5.7)	0 (0.0)	

Significant p-values are presented in bold.

### Mortality and re-hospitalization incidence

**Figure 1:**
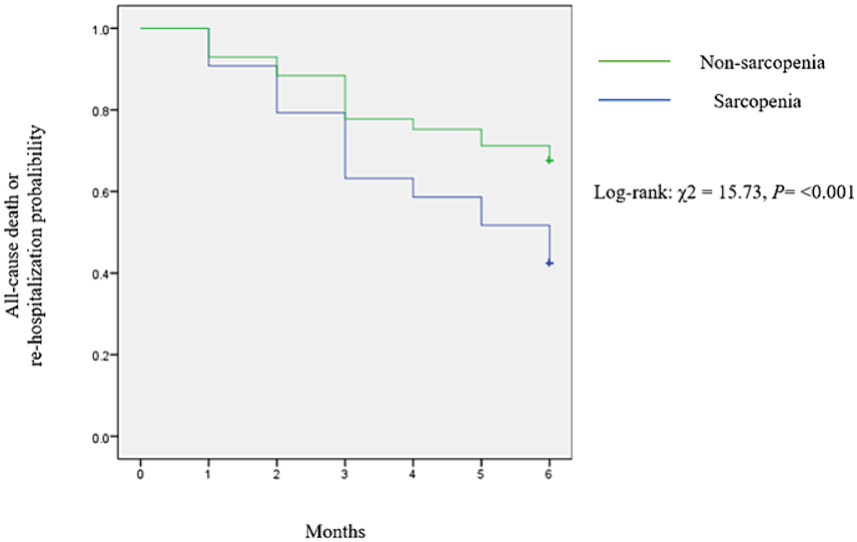
Kaplan–Meier curves illustrated all-cause death and all-cause re-hospitalization events stratified by SARC-F score.

During the 6-month follow-up period, there were 14 events (4.91%) of all-cause mortality and 100 events (35.08%) of all-cause re-hospitalization among the study participants. The total combined events, comprising both all-cause death and all-cause re-hospitalization, were 114, resulting in an incidence rate of 0.8 events per person-year. The Kaplan–Meier survival curves (Figure 1) illustrated a notably poorer prognosis for patients in the sarcopenia group compared to the non-sarcopenia group (*χ*^2^ = 15.73; log-rank test, *p* < 0.001).

The univariate analysis revealed that the hazard ratio (HR) for all-cause death and re-hospitalization events in patients with SARC-F score ≥ 4 compared to those with SARC-F score < 4 was 2.02 (95% CI: 1.39–2.92, *p* < 0.001) in the unadjusted analysis (Table 3).

**Table 3:** The hazard ratio for all-cause death and re-hospitalization in univariate analysis.

All-cause death and re-hospitalization events	HR	95% CI	*p*-value
SARC-F ≥ 4 (vs. SARC-F < 4)	2.02	1.39–2.92	<0.001

HR, Hazard ratio, CI, Confidence intervals.

During multivariate regression, sarcopenia (SARC-*F* ≥ 4), along with age, gender, diabetes, and heart failure, were included in the analysis. Notably, only sarcopenia indicated statistical significance. For individuals with SARC-F scores of 4 or higher compared to those with scores below 4, the HR is 1.66, with a 95% CI ranging from 1.06 to 2.59 (Table 4).

**Table 4:** The hazard ratio for all-cause death and re-hospitalization in multivariate analysis.

All-cause death and re-hospitalization events	HR	95% CI	*p*-value
SARC-F ≥ 4 (vs. SARC-F < 4)	1.66	1.06–2.59	0.026
Age	1.02	0.99–1.04	0.193
Female	0.83	0.56–1.22	0.346
Diabetes	1.42	0.98–2.06	0.065
Heart failure	1.41	0.93–2.13	0.105

HR, Hazard ratio, CI, Confidence intervals.

## Discussion

This study aimed to investigate the association between SARC-F scores and mortality risk in elderly patients with cardiovascular disease. The results of this study revealed that a SARC-F score of ≥4 was significantly associated with a higher risk of all-cause death and re-hospitalization events in this population.

This study observed that the number of patients with CVD having SARC-*F* ≥ 4 increased with age, was higher in females and in patients with heart failure or diabetes. The aging process encompasses various factors, such as natural declines in muscle mass and strength and increased inflammation, potentially contributing to the onset of sarcopenia (13). Besides, cardiovascular diseases, especially heart failure, may exacerbate the process of muscle loss (14). Chronic illnesses, especially CVD, can contribute to a state of systemic inflammation and metabolic changes that negatively affect muscle health (14). The combination of aging and the presence of CVD may induce or accelerate progression of sarcopenia. In addition, the most prominent pathway associated with sarcopenia and CVDs is insulin resistance. This phenomenon serves as a significant cardiovascular risk factor, independent of other risk factors, among older adults in community populations and individuals with diabetes (14). In a previous meta-analysis, Feng et al. (15) affirmed that sarcopenia was more prevalent in patients with diabetes. In accordance with our finding, Kitamura et al. (16) recorded that sarcopenia was more common in women with CVD.

Our study revealed a significant correlation between higher SARC-F scores and the presence of comorbidities. Certain comorbidities, such as diabetes, coronary heart disease, and vision problems, were identified as predictors of lower muscle strength in individuals aged 50 and older (17). Additionally, muscle mass and strength have been linked to elevated levels of inflammatory markers in patients with chronic diseases (13). Angulo et al. (18) found that multimorbidity at baseline was associated with a higher risk of sarcopenia during a twelve-year follow-up. Similarly, a systematic review by Pacifico et al. reported that individuals with dementia, diabetes, and respiratory diseases had a notably higher prevalence of sarcopenia compared to those without these conditions (19). Sarcopenia shares many risk factors with CVD, dementia, diabetes, and respiratory disease, such as sedentary behavior, low physical activity, inflammation, malnutrition, and various other mechanisms. This shared risk profile may explain the higher prevalence of sarcopenia in individuals with these age-related diseases (19). Consequently, there is a critical need to raise awareness and implement preventative strategies targeting both sarcopenia and its associated comorbidities.

Our findings suggest that a SARC-F score of 4 or higher is a predictor of a worse prognosis, including readmission or mortality post-discharge, in patients with CVD compared to those in the non-sarcopenia group (a score below 4). The identification of sarcopenia using the algorithm proposed by the European Working Group on Sarcopenia in Older People (EWGSOP), Yang et al. reported that this situation was associated with mortality during hospital stay and 1-year post-discharge among hospitalized older adults (7). A previous study by Ueshima et al. (20), a SARC-*F* ≥ 4 score was a predictor of death within 30 days of hospitalization. Takumi Noda et al. found that sarcopenia assessment using the SARC-F was associated with increased in-hospital mortality in older patients, as well as heightened short-term mortality in individuals with CVD (11). Sarcopenia diminishes both muscle mass and strength, potentially impairing balance and increasing the risk of falls, consequently increasing the likelihood of hospitalization (21). The reduced activity and prolonged bed rest associated with hospital stays can further exacerbate muscle mass and strength, worsening functional deterioration and increasing the probability of post-discharge falls and readmissions (22). This perpetuating cycle of functional decline and re-hospitalization may contribute to mortality (22). In fact, the pathogenesis of sarcopenia and cardiovascular diseases (CVDs) involves a complex interaction of multiple factors, including malnutrition, physical inactivity, insulin resistance, inflammation, hormonal changes (14). CVDs can exacerbate the adverse outcomes of sarcopenia, such as falls, fractures, hospitalization, and mortality (14). Conversely, sarcopenia significantly contributes to adverse outcomes in older individuals with CVDs. For instance, in patients with coronary heart disease, sarcopenia could predict poor outcomes in elderly patients undergoing percutaneous coronary intervention (PCI). In a study involving 475 elderly patients with coronary artery disease who underwent successful PCI, sarcopenia was assessed by measuring the cross-sectional area of skeletal muscle at the first lumbar vertebra (L1) (23). The findings revealed that 29.7% of patients had a low L1 skeletal muscle index, which independently predicted all-cause mortality and major adverse cardiovascular events (23). Therefore, early identification and diagnosis of sarcopenia in primary care settings and hospitals are vital for initiating preventive or intervention strategies, thus mitigating the risks associated with sarcopenia and reducing the overall healthcare burden and expenses.

Sarcopenia is not merely a reduction in muscle mass but reveals significant implications for functional abilities. This study revealed an association between higher SARC-F scores and poorer functional outcomes. It suggests that individuals with sarcopenia may face challenges related to strength, mobility, and performing daily activities, indicating the importance of assessing this phenomenon clinically. In the African American Health (AAH) cohort, participants with SARC-F scores ≥4 exhibited slower chair stand times and weaker grip strength (5). Similarly, in the NHANES 1999–2006 survey, individuals with SARC-F scores ≥4 demonstrated slower walking times and weaker knee extension strength compared to the control group (5). These findings emphasize the clinical relevance of sarcopenia assessment and underscores the value of tools like SARC-F in recognizing and addressing this condition in elderly populations and those with cardiovascular disease. By identifying individuals at risk of sarcopenia and understanding its impact on functional abilities, healthcare providers can implement appropriate interventions to improve outcomes and quality of life for these individuals.

It is worth noting that this study has some limitations. Firstly, the study was conducted at a single tertiary hospital, which may limit the generalizability of the findings to other settings. Secondly, the responses to the SARC-F questionnaire by some patients may have been influenced by undetected, subtle, transient cognitive impairments associated with their acute condition. Thirdly, the exclusion criteria applied, including acute severe illness and pacemaker implantation, may have inadvertently excluded certain relevant patient populations. Furthermore, the study did not extensively consider potential confounding factors that could influence the association between SARC-F scores and adverse outcomes, such as medication use, socioeconomic status, cognitive function, nutritional status and lifestyle habits. Finally, the study lacked a control group of younger individuals for comparison of SARC-*F* values between older and younger subjects.

Despite such limitations, the study addresses a gap in the context of dementia and its risk factors among Vietnamese people. This study has major strengths by addressing a gap in the literature regarding the role of the SARC-F questionnaire for predicting risk of adverse outcomes among Vietnamese elderly patients with cardiovascular disease. The study by Shinya Tanaka et al. has indicated that combining physical function measures with the SARC-F questionnaire might enhance predictive accuracy in elderly patients with CVD upon admission. This combined approach did not result in a statistically significant difference when compared to using the SARC-F questionnaire alone (24). These findings suggest that in clinical settings where time constraints limit the feasibility of conducting extensive physical function assessments, the SARC-F questionnaire should be a recommended and practical tool for prognostic evaluation among this patient population.

The study’s findings have important clinical implications. Findings from the study sheds light on the prognostic value of the SARC-F questionnaire in identifying elderly patients at risk of adverse outcomes in the cardiovascular setting. In the context of geriatric cardiovascular care, the study emphasizes the need for comprehensive geriatric assessment tools, such as the SARC-F questionnaire, to identify vulnerable patients who may benefit from tailored interventions and closer monitoring. The SARC-F questionnaire can be easily administered in a clinical setting, making it a valuable tool for identifying elderly CVD patients at a higher risk of adverse outcomes. Early identification of these high-risk patients can help healthcare providers implement appropriate interventions and strategies to improve patient outcomes and reduce the burden of re-hospitalization. Integrating sarcopenia screening into routine cardiovascular evaluations can enhance risk stratification and guide personalized treatment strategies aimed at optimizing outcomes in older adults with cardiovascular conditions. Moreover, raising awareness among healthcare providers about the importance of assessing sarcopenia in elderly patients with cardiovascular disease could facilitate early identification and intervention.

Future prospective studies employing more age groups, larger sample sizes, and a comprehensive panel of risk factors in a multi-center clinical trial can provide further insights into the predictive utility of SARC-F for adverse outcomes in the elderly.

## Conclusion

Among elderly patients with CVD, the SARC-F questionnaire is a valuable tool for predicting mortality and the risk of re-hospitalization in elderly patients with cardiovascular disease. A SARC-F score of ≥4 was associated with a significantly higher risk of all-cause death and re-hospitalization. The SARC-F questionnaire offers a simple and cost-effective method for screening and prognostic evaluation in busy clinical settings. The study sheds light on the potential of the SARC-F questionnaire as a screening tool for predicting adverse outcomes in elderly patients with cardiovascular disease. Future multi-center trials with diverse age groups, larger samples, and comprehensive risk factor panels can provide deeper insights into SARC-F’s predictive value for adverse outcomes in older adults.

## Data availability statement

The raw data supporting the conclusions of this article will be made available by the authors, without undue reservation.

## Ethics statement

The studies involving humans were approved by Ethical Committee of the University of Medicine and Pharmacy at Ho Chi Minh City (Number: 544/HĐĐĐ-ĐHYD, signed September 22, 2022). The studies were conducted in accordance with the local legislation and institutional requirements. Written informed consent for participation in this study was provided by the participants’ legal guardians/next of kin.

## Author contributions

TaN: Conceptualization, Writing – original draft, Writing – review & editing, Data curation, Investigation, Project administration, Software, Validation. TN (2nd author): Conceptualization, Investigation, Software, Writing – original draft, Writing – review & editing. HC: Data curation, Methodology, Writing – original draft, Writing – review & editing. TN (4th author): Supervision, Writing – original draft, Writing – review & editing. TrN: Formal analysis, Project administration, Writing – original draft, Writing – review & editing. DD: Project administration, Validation, Writing – original draft, Writing – review & editing. TL: Validation, Writing – original draft, Writing – review & editing.
